# Toward improved terpenoids biosynthesis: strategies to enhance the capabilities of cell factories

**DOI:** 10.1186/s40643-022-00493-8

**Published:** 2022-01-24

**Authors:** Eric Fordjour, Emmanuel Osei Mensah, Yunpeng Hao, Yankun Yang, Xiuxia Liu, Ye Li, Chun-Li Liu, Zhonghu Bai

**Affiliations:** 1 National Engineering Laboratory for Cereal Fermentation Technology, Jiangnan University, 1800 Lihu Road, Wuxi, 214122 Jiangsu China; 2 Jiangsu Provincial Research Centre for Bioactive Product Processing Technology, Jiangnan University, Wuxi, China

**Keywords:** Terpenoids, Protein engineering, Dynamic regulation, Promoter engineering, RBS engineering, Cellular tolerance, Chromosomal integration, Modular engineering

## Abstract

Terpenoids form the most diversified class of natural products, which have gained application in the pharmaceutical, food, transportation, and fine and bulk chemical industries. Extraction from naturally occurring sources does not meet industrial demands, whereas chemical synthesis is often associated with poor enantio-selectivity, harsh working conditions, and environmental pollutions. Microbial cell factories come as a suitable replacement. However, designing efficient microbial platforms for isoprenoid synthesis is often a challenging task. This has to do with the cytotoxic effects of pathway intermediates and some end products, instability of expressed pathways, as well as high enzyme promiscuity. Also, the low enzymatic activity of some terpene synthases and prenyltransferases, and the lack of an efficient throughput system to screen improved high-performing strains are bottlenecks in strain development. Metabolic engineering and synthetic biology seek to overcome these issues through the provision of effective synthetic tools. This review sought to provide an in-depth description of novel strategies for improving cell factory performance. We focused on improving transcriptional and translational efficiencies through static and dynamic regulatory elements, enzyme engineering and high-throughput screening strategies, cellular function enhancement through chromosomal integration, metabolite tolerance, and modularization of pathways.

**Graphical Abstract:**

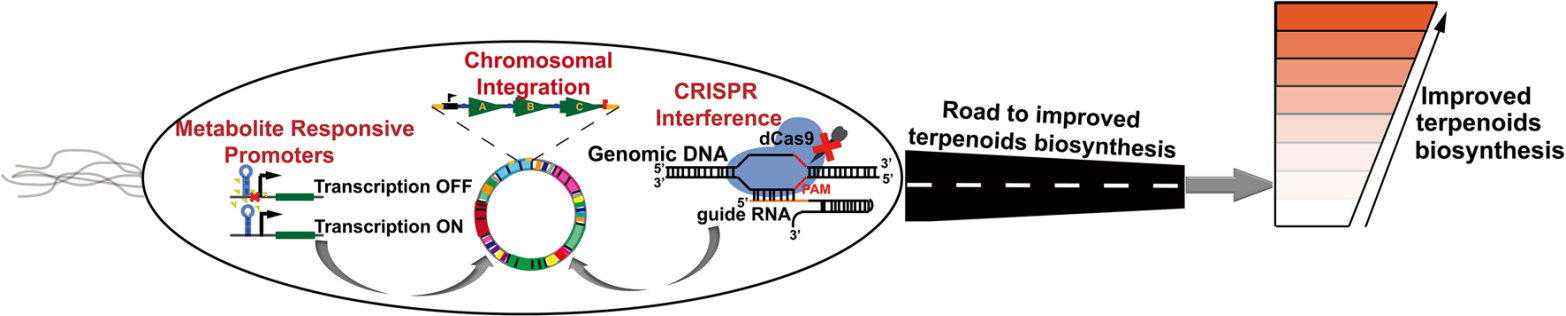

## Introduction

**Fig. 1:**
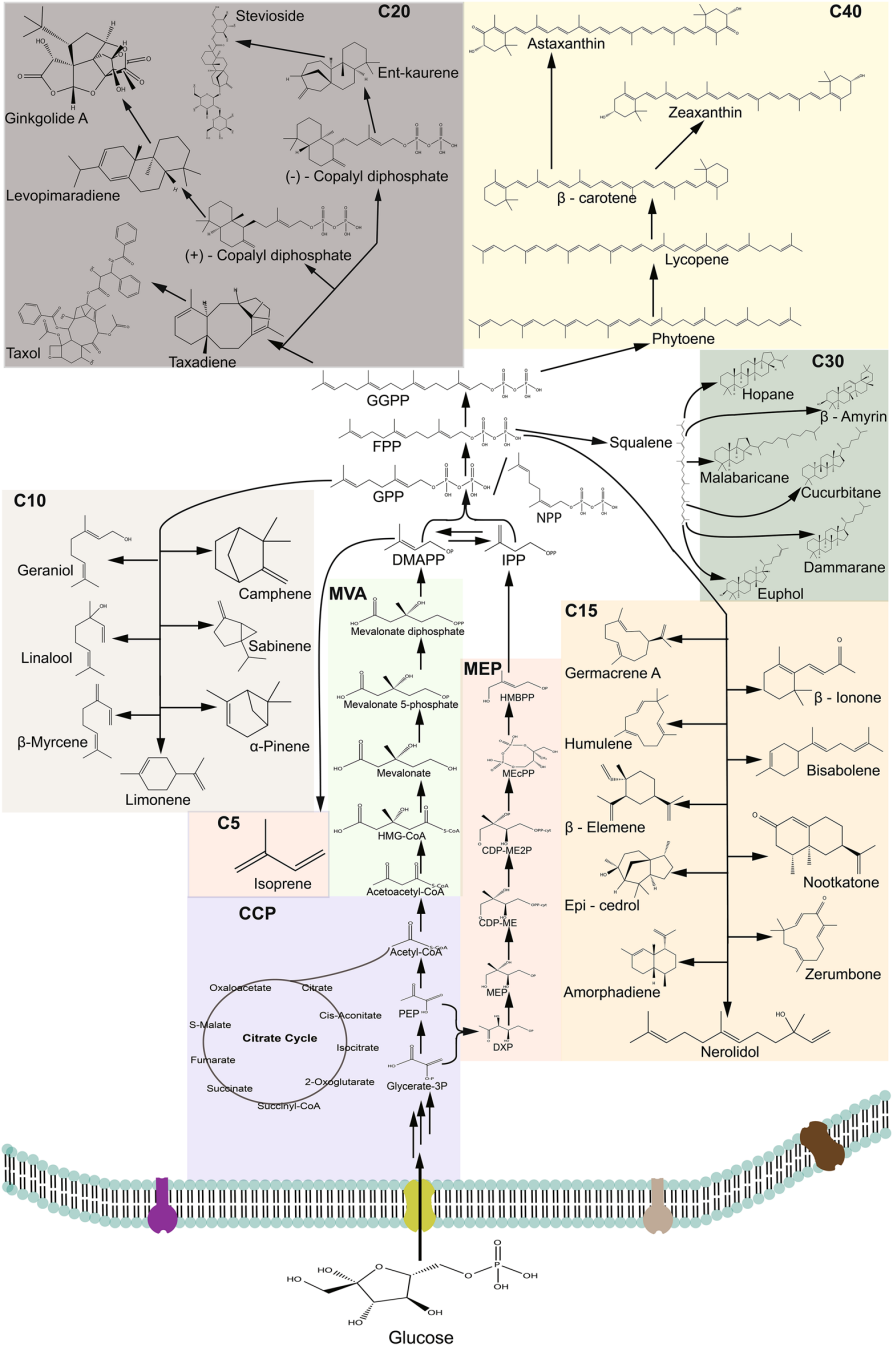
The MVA pathway and MEP pathway for isoprenoids biosynthesis. The isoprenoid biosynthetic pathway can be grouped into the central carbon pathway, upstream isoprenoid pathway, and downstream isoprenoid pathway. PEP, phosphoenolpyruvate; HMG-CoA, S-3-hydroxy-3-methylglutaryl-CoA; DXP, 1-deoxy-d-xylulose 5-phosphate; MEP, 2-C-methyl-d-erythritol 4-phosphate; CDP-ME, 4-(cytidine 5ʹ-diphospho)-2-C-methyl-d-erythritol; CDP-ME2P, 2-phospho-4-(cytidine 5ʹ-diphospho)-2-C-methyl-d-erythritol; MEcPP, 2-C-methyl-d-erythritol 2,4-cyclodiphosphate; HMBPP, 1-hydroxy-2-methyl-2-butenyl 4-diphosphate; IPP, isopentenyl diphosphate; DMAPP, dimethylallyl diphosphate; GPP, geranyl pyrophosphate; NPP, neryl pyrophosphate; FPP, farnesyl pyrophosphate; GGPP, geranylgeranyl pyrophosphate; CCP, central carbon pathway; C5, hemiterpenoids; C10, monoterpenoids; C15, sesquiterpenoids; C20, diterpenoids; C30, triterpenoids; C40, tetraterpenoids

Terpenoids form the most complex class of natural products in chemical and structural terms (Gershenzon and Dudareva 2007; McGarvey and Croteau 1995). Terpenoids (isoprenoids) constitute over 80,000 identified natural compounds, making them the largest known natural compounds (Tetali 2019; Tholl 2015). Terpenoids have a wide distribution in plants, microorganisms, insects, and marine invertebrates (Bian et al. 2018; Chen et al. 2011; Huber et al. 2015; Yamada et al. 2015). Plants exhibit a vast array of isoprenoids; hemiterpenoids (C_5_), monoterpenoids (C_10_), sesquiterpenoids (C_15_), diterpenoids (C_20_), sesterterpenoids (C_25_), triterpenoids (C_30_), and tetraterpenoids (C_40_) (Fig. 1). These naturally occurring products can be harnessed into useful compounds in the pharmaceutical, food, agricultural, and chemical industries due to their many different properties. Terpenoids are derived from the five-carbon (C5) intermediary units isopentenyl diphosphate (IPP) and its double-bond isomer dimethylallyl diphosphate (DMAPP) from two major universal pathways: the mevalonate (MVA) pathway and the 2-C-methyl-d-erythritol 4-phosphate (MEP) pathway. These are subsequently converted into the plethora of isoprenoids by their respective prenyltransferases and terpene synthases (Tholl 2015) (Fig. 1). This has led to the synthesis of numerous drugs, health care, cosmetic products, flavor and fragrant agents, and biofuels (Tetali 2019).

Given their potential benefits in various sectors, the demand for terpenoids is rapidly increasing. Low yields and high costs restrict the direct extraction from plants and other naturally occurring sources. Synthetic production, an alternative source for terpenoid synthesis, produces several isomers. These synthetic chemicals come with several health issues that restrict their direct application due to the possible transfer of by-products and intermediates. However, “wild-type” microbes are unsuitable for commercial purposes since they produce low yields. Designing ideal industrial cell factories is an alternative to producing terpenoids and their derivatives that meet safety and economic concerns. Eliminating the negative feedbacks in the precursor pathways is one of the strategies to increase the pool of precursors. Since wild-type strains are inefficient for industrial applications, it is therefore expedient to metabolically engineer them for high-producing strains.

To ensure a smooth transition to a bio-based economy devoid of complications associated with natural and artificial biosynthetic pathways, developing new, versatile, industrial microbial platforms is required (Jakočiūnas et al. 2020). Since cells have extensive metabolic networks with hard-wired, strictly regulated molecular pathways that resist resource redirection, turning them into successful factories is difficult. (Nielsen and Keasling 2016). The emergence of synthetic biology has facilitated the production of chemicals that, in the past, could not be produced in desired hosts by simply expressing and fine-tuning exogenous pathway genes. To boost productivity, metabolic pathways in these production hosts are frequently regulated by several genetic regulatory tools (Fordjour et al. 2019; Ward et al. 2018). Nevertheless, microbial metabolism is extremely complicated that achieving sufficient yield of a target product from engineered microbe demands careful studies and understanding, as well as the availability of essential tools to manipulate the host strain. The expression of heterologous genes normally results in a metabolic burden on the host strain, affecting the yield of target products. The goal of synthetic biology is to create predictable biological systems. However, bacteria, for example, are very complicated organisms, making it tough to comprehend all of the cell’s functions at the same time and to predict the perturbation outcomes (Jervis et al. 2019).

To overcome these metabolic hurdles and increase the production of metabolites, several strategies have been adopted including: (i) DNA-based engineering, for example, promoter engineering; (ii) RNA engineering including synthetic RNA switches; (iii) protein and cofactor engineering; (iv) metabolic pathway engineering, including modular pathway engineering and compartmentalization engineering; (v) genome-wide engineering; (vi) cell engineering, including transporter engineering (Chen et al. 2018b). This review analyzes recent progress in microbial biosynthesis of terpenoids and examines the critical issues and challenges confronting engineering of cell factories for commercial purposes. In this review, we summarize recent synthetic biology and metabolic engineering strategies to address these challenges and carefully construct a suitable chassis for industrial purposes. We focused on how transcriptional and translational efficiencies through static and dynamic regulatory elements have been harnesses for cell factory development. Also, enzyme engineering and high-throughput screening strategies, cellular function enhancement through chromosomal integration, cell tolerance, and modularization of pathways have been discussed here.

## Strategies for developing cell factories for terpenoid biosynthesis

### Protein engineering

The lack of a centralized database with adequate functionally annotated sequence data has impeded a thorough study of terpene synthases and prenyltransferases in terms of product specificity. In the metabolic process of terpenoid synthesis, terpenoid synthases and prenyltransferases catalyze the synthesis of biologically important terpenoid compounds (Keeling and Bohlmann 2006) (Fig. 1). As is especially true in the case of the terpenoid pathway, various features of a metabolic pathway are not associated exclusively with increasing the concentration of enzymes. Enzymes in their natural states do not show the stability, specificity, or catalytic efficiency required for particular processes (Kokkonen et al. 2019). Protein engineering has been successfully applied to optimize the catalytic efficiencies of rate-limiting enzymes. This has happened through directed evolution and structure-guided engineering using structural information as a tool for enzyme engineering. The lack of a thorough understanding of the structure/function relationship of these terpene synthases and prenyltransferases becomes a deficiency in how to engineer them for high isoprenoids production. To improve properties, protein engineering techniques such as de novo design, directed evolution, rational design, and analytical techniques can be used (Fig. 4). To ensure efficient screening of variants, several high-throughput screening approaches have been developed (Zeng et al. 2020). A lycopene-dependent color high-throughput screening method has already been developed. Adopting this screening strategy, an improved variant form of IDI bearing triple-mutation (L141H/Y195F/W256C) with a catalytic activity of 2.53-fold higher than the wild-type was selected after a directed evolution and site-saturation mutagenesis process. The final strain expressing the mutated enzyme produced more than 1.2 g/L of lycopene, a 2.8-fold increment as compared to the wild-type (Chen et al. 2018a). Wang et al. also developed a novel high-throughput screening method based on DMAPP toxicity to screen for enhanced isoprene synthases (ISPS). Error-prone PCR was used to generate ISPS variants that were cloned into an already constructed DMAPP high-producing strain. A combinatorial mutant with a double mutation (A570T/F340L) was developed to produce isoprene threefold higher than the wild-type strain (Wang et al. 2017).

**Fig. 2:**
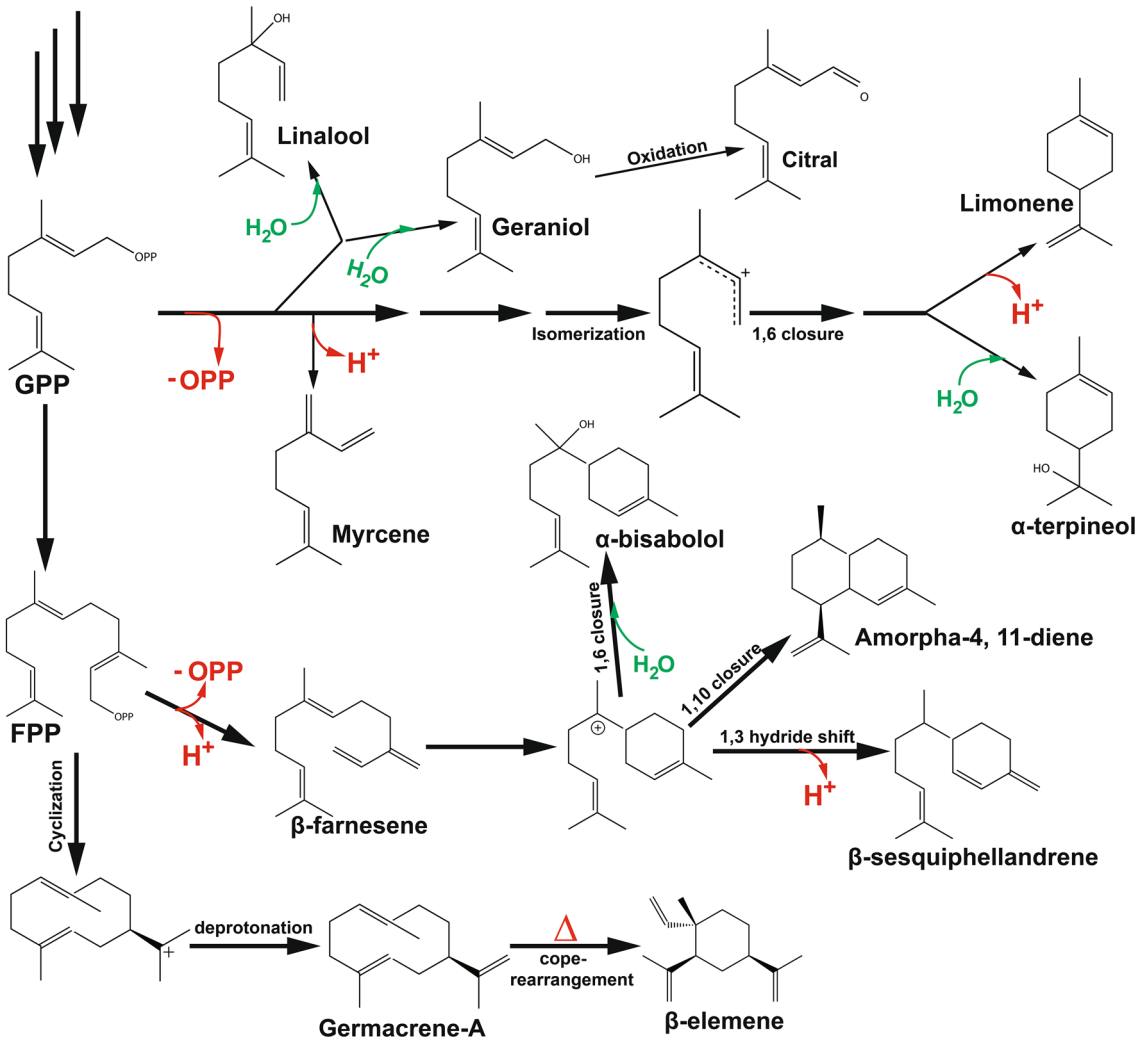
Proposed mechanism of terpenoid carbonation. Terpenoids undergo a wide range of cyclization and rearrangements to ensure final product synthesis

Terpenoid synthases are known to generate intermediates in enzyme-bound carbocation, to achieve structural and functional diversity. This happens after they go through a series of reconfigurations and carbocation quenching (Fig. 2). The process of enzyme carbocation is very important when dealing with protein engineering and has been extensively highlighted by Hong et al. (2020), Huang et al. (2021), Ker et al. (2020), Leferink et al. (2019), McClelland (2008), Raz et al. (2020), Salmon et al. (2015), Tantillo (2010, 2017). Readers are therefore urged to consult the suggestions for further reading. Such functional promiscuity is associated with poor catalytic properties and undesirable product creation (Nobeli et al. 2009). Though enzyme promiscuity is known to provide organisms with genome plasticity to thrive in extreme environmental conditions by altering and reprogramming their metabolic pathways or suppression of undesirable activity (Guzmán et al. 2019), these cyclization reactions often produce “impure” compounds with undesirable products. This increases the cost of production as undesirable by-products have to be removed to ensure a clean commercial product. But the unavailability of enough information about cyclization type and active site sequence (Chen et al. 2011; Christianson 2006) and a holistic and predictive understanding of structural and stability hampers the use of rational engineering to build models of proteins with desirable properties for this purpose. Levopimaradiene (LP), a diterpenoid, is a metabolic product of Levopimaradiene synthase (LPS) via its complex reaction cascade of cyclization, rearrangement, and proton transfers using geranylgeranyl pyrophosphate (GGPP) as its substrate. LPS is known for its promiscuity as it produces isomeric side products such as abietadiene, sandaracopimaradiene, and neoabietadiene (Peters et al. 2000; Ravn et al. 2000). A combinatorial mutation engineering was used to screen for LPS variants with enhanced diterpenoid productivity and selectivity towards LP. The final strain produced an approximately 2600-fold increase in LP. An approximate 700 mg/L of LP was produced in a bench-scale bioreactor under a controlled condition (Leonard et al. 2010).

Taxol (paclitaxel) has been a potent chemotherapeutic drug in recent years. However, microbial production is normally hampered by the decomposition of its pathway intermediates to produce undesired products as less than 10% of taxadiene has been speculated to be converted to taxadien-5α-ol by the taxadiene-5-α-hydroxylase (CYP725A4). To ensure product specificity and reduce by-product formation, an alternative pathway was created by mutating taxadiene synthase through site-saturated mutagenesis to yield a 2.4-fold improvement in taxa-4(20)-11(12)-diene which was subsequently hydroxylated to taxadien-α5-ol by CYP725A4 (Edgar et al. 2017). Another strategy for improving protein activity is via enzyme fusion. The fusion of protein has gained increasing recognition in creating novel protein therapeutics and enhancing the performance of engineered strains for biomolecules synthesis. Enzyme fusion is a technique of joining enzymes to produce a recombinant protein with combined characteristics of parental proteins (Uhlen et al. 1992). Enzyme fusion has found application in various areas of biotechnology such as protein purification (Terpe 2003), imaging (Yuste 2005), biopharmaceuticals (Berger et al. 2015), and facilitating downstream fermentation processes (Uhlen et al. 1992). Several reasons for constructing these artificially fused enzymes include improved catalytic activity, activated substrate channeling due to proximity to biocatalysts, higher stability, and cheaper production processes (Elleuche 2015).

**Fig. 3:**
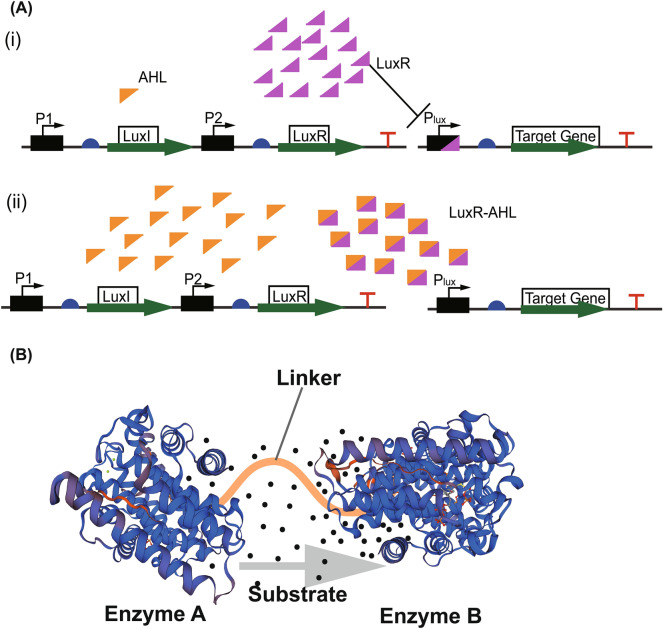
**A** Illustration of dynamic control of LuxI/LuxR Quorum Sensing (QS) system. **(i)** At low cell density, the transcriptional regulator, LuxR, binds the P_lux_ promoter to repress the transcription of the target gene. **(ii)** At high cell density, LuxI protein synthesizes acyl-homoserine lactone (AHL) which binds the transcriptional regulator resulting in dissociation from the promoter. The target gene is subsequently expressed. **B** Graphical representation of putative enzymes joined with a linker. Sequential pathway enzymes can be modified through enzyme fusion to improve enzymatic reaction. The fusion of enzymes ensures substrates are channelled from one active site to the other

In metabolic engineering, one of the main objectives of fusing enzymes is to ensure active sites are close to ensuring intermediates are channeled from active site A to active site B while also preventing competition for these intermediates (Fig. 3B). The direct fusion of enzymes without spacers or linkers, which are indispensable components in building stable, bioactive fused proteins, could result in poor protein expression and reduced catalytic activity (Chen et al. 2013b). Hence, to maintain the functionality of fused enzymes, linkers or spacers are needed. Biosynthesis of isoprene is gaining enough ground because of its associated commercial application (Liu et al. 2019). To increase isoprene production in Cyanobacteria, an isoprene synthase was fused with a highly expressed native protein, *cpcB*, with or without a linker. A strain with a seven amino acid linker generated 28.9 µg/L/h of isoprene, a 27-fold higher than the strain bearing the unfused enzymes. The study concluded that the relative folding of two enzymes with respect to one another facilitates their catalytic activity (Chaves et al. 2017). Therefore, linkers are an important component in recombinant fusion protein technology. The close active sites of fused proteins guarantee substrate utilization, prevent intermediate diffusion, and alleviate feedback inhibition (Dale et al. 2003). Enzyme fusion has also been proven to remove competition for substrates (Camagna et al. 2019). In *S. cerevisiae*, geranyl pyrophosphate (GPP), the primary precursor for monoterpenes production, is also a precursor for FPP production. To improve the availability and efficient utilization of GPP for geraniol production, Erg20^WW^ was fused with truncated geraniol synthase in both forward and reverse form. There was 15% increment in geraniol production after 120 h of fermentation compared to the unfused strain (Jiang et al. 2017).

Notable properties of these linkers like secondary structure, length, hydrophobicity, amino acid composition, protease sensitivity, and potential interactions with fused proteins are essential determinants in enzyme fusion technology (Yu et al. 2015). The synthesis of carotenoids begins with the production of phytoene from GGPP catalyzed by phytoene synthase (*crtB*). Phytoene desaturase (*crtI*) then catalyzes the synthesis of lycopene, which is finally cyclized to form carotenoids by the lycopene cyclase (*crtY*). To facilitate an improved production of carotenoid, *crtI* and *crtB* domains were joined using two possible configurations with natural and synthetic linkers. A tridomain enzyme comprising *crtB*, *crtI*, and *crtY* was also created. The results from this study demonstrated that the domain order and linker properties affect the expression and stability of the fused proteins, which affects their catalytic effects (Rabeharindranto et al. 2019). In practice, directed evolution, site-directed evolution, enzyme truncation, the fusion of enzymes with other enzymes and or tags are suitable strategies to improve the activities of enzymes. Remarkable advances have been made in enzyme restructure and modification via protein engineering. These could be found in Table 1. This prevents the accumulation of precursors that have resulted in host toxicity. However, the lack of structural–functional relationships that accompanies the fused enzymes becomes a significant challenge as fused enzymes may encounter misfolding, impaired catalytic activity, and low expression level, affecting their desired intentions. Investigations are required to decipher domain–linker interactions and domain–domain interactions to advance the designing of suitable cell factories.

**Table 1:** Typical protein engineering strategies for terpenoid biosynthesis

Strain	Product	Description	Outcome	Refs.
Protein engineering				
* E. coli*	Viridiflorol	Random mutation by error-prone PCR and enzyme truncation on viridiflorol synthase	25.7 g/L	Shukal et al. (2019)
* E. coli*	Levopimaradiene	Combinatorial evolution in geranylgeranyl diphosphate synthase and levopimaradiene synthase	700 mg/L	Leonard et al. (2010)
* E. coli*	Taxa-4(20)-11(12)-diene	Site-saturated mutagenesis on taxadiene synthase	2.4-fold	Edgar et al. (2017)
* E. coli*	Amorpha-4,11-diene	Mutability landscape engineering of amorpha-4,11-diene synthase	Fourfold	Abdallah et al. (2018)
* E. coli*	Acetyl-CoA	Site-directed mutagenesis of citrate synthase (*gltA*)	0.24 g/g glucose	Tovilla-Coutiño et al. (2020)
* E. coli*	8-*epi*-cedrol	Fusion of farnesyl pyrophosphate and Epi-cedrol synthase	1.084 mg/L	Navale et al. (2019)
* E. coli*	Astaxanthin	Fusion of *crtZ* and *crtW*	1.4-fold increment	Nogueira et al. (2019)
* E. coli*	Steviol glycosides	Fusion of *CYP714A2* and P450 reductase 2 (*AtCPR2*) from *A. thaliana*	38.4 ± 1.7 mg/L	Moon et al. (2020)
* E. coli*	Lycopene	Enzyme engineering of mevalonate 3-kinase via single amino acid mutations into 5-phosphomevalonate 3-kinase	N. A	Motoyama et al. (2019)
* E. coli*	Albaflavenol and (1R)-6β-hydroxycineole	Fusion of terpene synthases and cytochrome P450	90- and 5.4-fold improvement, respectively	Wang et al. (2021d)
* E. coli*	Geraniol	Fusion tagging of geraniol synthase with a laboratory evolved N-terminal of a chloramphenicol acetyltransferase leader sequence	2124.1 mg/L	Wang et al. (2021c)
* E. coli*	(−)-Carvone	Optimization of pathway enzymes with quantification concatemer (QconCat) method	15-fold	Yoshida et al. (2021)
* E. coli*	Pinene	Computer-aided directed evolution of geranyl diphosphate and pinene synthase	N. A	Chen et al. (2021)
* E. coli*	Nerol and borneol	Site-directed mutagenesis of truncated bornyl diphosphate synthase from *Lippia dulcis*	966.55 mg/L and 87.20 mg/L, respectively	Lei et al. (2021)
* E. coli*	(1R)-6β-Hydroxycineole and albaflavenone	Fusion of 1,8-cineole synthase and cytochrome P450 enzyme CYP176A1 for as well as fusion of epi-isozizaene synthase with CYP170A1	5.4-, 2.3-fold, respectively	Wang et al. (2021d)
* E. coli*	Isopentenol	Improved activity of phosphomevalonate decarboxylase (PMD) in an isopentenyl diphosphate (IPP)-bypass	2.4-fold	Kang et al. (2017)
* S. cerevisiae*	Geranylgeraniol	Overexpression of fused geranylgeranyl pyrophosphate synthase from *Pantoea agglomerans* and farnesyl diphosphate synthase (*PaGGPPS-Erg20*) and fused geranylgeranyl pyrophosphate synthase from *Pantoea agglomerans* and diacylglycerol diphosphate phosphate (*PaGGPPS-DPP1*)	5.07 g/L	Wang et al. (2021b)
* S. cerevisiae*	Miltiradiene	Engineering a chimeric protein through the fusion of terpene synthase 1 from *Coleus forskohlii* and kaurene synthases-like from *Salvia miltiorrhia*	3.5 g/L	Hu et al. (2020a)
* S. cerevisiae*	Isoprene	Directed evolution of isoprene synthase	3.7 g/L	Wang et al. (2017)
* S. cerevisiae*	Lycopene	Inactivation of lycopene cyclase activity of *crtYB* via directed evolution and improved activity of *crtE*	1.61 g/L	Xie et al. (2015)
* S. cerevisiae*	Lycopene	Directed evolution on isopentenyl diphosphate isomerase (*idi*)	1.2 g/L	Chen et al. (2018a)
* S. cerevisiae*	Lycopene and β-carotene	Screening of efficient crt variants from *Sporidiobolus pararoseus* TBRC-BCC 63403	Four and sevenfold, respectively	Watcharawipas et al. (2021)
* S. cerevisiae*	Carotenoid	A bidomain fusion of *crtI* and *crtB* and a tridomain fusion of *crtI*, *crtB*, and *crtY*	Twofold	Rabeharindranto et al. (2019)
* S. cerevisiae*	α-farnesene	Screening of an α-farnesene synthase with enhanced catalytic activity	10.4 g/L	Wang et al. (2021a)
* S. cerevisiae*	d-Limonene	Fusion of truncated limonene synthase from *Citrus limon* and truncated neryl diphosphate synthase	23.7 mg/L	Hu et al. (2020b)
* S. cerevisiae*	Diterpenoids	Fusion of Erg20^WW^, Erg20 and BTS1	3.2- and 2.3-fold	Dong et al. (2020a)
* S. cerevisiae*	Geraniol	Fusion of Erg20^WW^ and geraniol synthase	15% improvement	Jiang et al. (2017)
* S. cerevisiae*	Germacrene A	Fusion of farnesyl pyrophosphate synthase and germacrene A synthase	190.7 mg/L	Hu et al. (2017)
* S. cerevisiae*	( +)-Nootkatone	Fusion of (+)-valencene synthase and farnesyl diphosphate synthase	59.78 mg/L	Meng et al. (2020a)
*S. cerevisiae*	Zerumbone	Fusion of cytochrome P450 reductase from *A. thaliana* and α-humulene 8-hydroxylase (*CYP71BA1*)	40 mg/L	Zhang et al. (2018a)
* S. cerevisiae*	Sabinene	N-terminal truncation of sabinene synthase	96% improvement	Jia et al. (2020)
* S. cerevisiae*	Linalool	N-terminal SKIK tagging of engineered linalool synthase and RIAD/RIDD linking of SKIK linalool synthase and ERG20^WW^	42% improvement	Zhou et al. (2021)
* S. cerevisiae*	Lutein	Localization of lycopene ε-cyclase to the cell membrane through fusion technology	N. A	Bian et al. (2021)
* S. cerevisiae*	Violaxanthin	N-terminal truncation of zeaxanthin epoxidases	7.3 mg/g DCW	Cataldo et al. (2020)
* S. cerevisiae*	α-terpineol	Fusion of truncated α-terpineol synthase with *Erg20*^*WW*^	21.88 mg/L	Zhang et al. (2019)
* Y. lipolytica*	(+)-nootkatoone	Enzyme fusion of truncated NADPH-cytochrome P450 reductase and (+)-nootkatoone synthase coupled with another optimization	20.5-fold improvement	Guo et al. (2018)
* Y. lipolytica*	Gibberellin (GA_3_)	Protein engineering and overexpression of pathway enzymes	12 mg/L	Kildegaard et al. (2021)
* Y. lipolytica*	α-farnesene	Fusion of *Erg20* and farnesyl synthase with lipid as the carbon source	10.2 g/L	Liu et al. (2021d)
* Pantoea ananatis*	Linalool	Improved soluble expression of (S)-linalool synthase by N-terminal fusion with halophilic β-lactamase hexahistidine	10.9 g/L	Nitta et al. (2021)
* C. glutamicum*	Astaxanthin	Fusion of membrane-bound *crtW* and *crtZ*	3.1 mg/g DCW	Henke and Wendisch (2019)
* Bacillus subtilis*	Amorphadiene	Site-directed mutagenesis of amorphadiene synthase with a one-plasmid CRISPR–Cas9 editing system	116 mg/L	Song et al. (2021)
Cyanobacteria	Isoprene	Fusion isoprene synthase with the native promoter *cpcB*	28.9 µg/L/h	Chaves et al. (2017)

### Dynamic pathway regulation

The use of static regulatory elements results in cellular perturbations which can only be addressed through a comprehensive fine-tuning of the various regulatory parts. Static regulation often results in metabolic imbalances that affect a cells’ productivity. Dynamic control of metabolic pathways is crucial in debugging bottlenecks at various points of enzymatic reactions. Taking cues from the natural regulatory metabolic network that respond to intracellular conditions, dynamic regulators have been engineered to manage the production of metabolites and cell growth (Holtz and Keasling 2010). Various dynamic regulatory mechanisms including global regulators (Farmer and Liao 2000), environmental cues (Harder et al. 2018; Yin et al. 2017; Zhao et al. 2018), and chemical cues (Ge et al. 2020) to regulate pathway expression have been developed to ensure a careful balance between the production of biomass and metabolites. Recently, a configurable responsive genetic circuit that genetically controls the activation and repression of pathway genes was developed to control intracellular pyruvate concentration (Xu et al. 2020). In a related study, Shen et al. (2016) adopting feedback responsive promoter enhanced the zeaxanthin synthesis by dynamically regulating the mevalonate pathway to prevent the accumulation of toxic precursors. To enhance the synthesis of monoterpenes, the Erg20 was degron-tagged to control the downstream flux which competes with GPP accumulation, the main precursor for monoterpenes synthesis (Peng et al. 2018).

Dynamic regulation, as an approach, helps microorganisms to thrive in changing environmental conditions and regulate homeostasis, and metabolic flux. One major known method for dynamically regulating pathways is found in the “two-staged metabolic control system”. This involves decoupling the growth and production stages into two to maximize biomass and the production of metabolites (Hartline et al. 2021). One of such regulatory mechanisms is quorum sensing (QS), a mechanism of cell-to-cell communication dependent on cell density in several species of microorganisms, particularly in bacteria (Papenfort and Bassler 2016). This intercellular communication enables bacteria to make a collective decision based on their population. The QS generates, releases, and detects auto-inducers at a certain threshold of cell density (Ge et al. 2020) (Fig. 3A). Knowing the mechanisms at the molecular level of this naturally occurring cell–cell communication system lays a foundation for the engineering of living cells to perform specified and unique tasks. Adopting the two-component QS system *luxI*–*luxR* from *Vibrio fischeri*, Kim et al., achieved 44% increment in bisabolene production from their previous work. In this work, seven variants of the sensor plasmid, carrying *luxI*–*luxR* genes, and four variants of the Response plasmid under the control of P_*luxI*_ promoter, carrying pathway genes to produce bisabolene, were designed to improve the biosynthesis of bisabolene. To avert the problems associated with the plasmid-associated pathway expression system, the QS-based bisabolene pathway was integrated into the *E. coli* strain resulting in a 1.1 g/L of bisabolene production (Kim et al. 2017). Examples of QS system-associated regulation of metabolic pathways for terpenoids production can be seen in Table 2.

**Table 2:** Dynamic and static regulatory strategies used to enhance the cell factory productivities

Strain	Product	Description	Outcome	Refs.
Dynamic regulation				
* E. coli*	Bisabolene	An inducer-free Lux QS system	1.1 g/L	Kim et al. (2018)
* E. coli*	Lycopene	Engineering the Ntr regulon to control intracellular metabolites	18-fold	Farmer and Liao (2000)
* E. coli*	Zeaxanthin	IPP/FPP-responsive promoter to regulate tuneable intergenic regions (TIGRs)	2.1-fold	Shen et al. (2016)
* S. cerevisiae*	Linalool and Limonene	An N-degron-dependent protein degradation strategy to downregulate Erg20p	18 and 76 mg/L, respectively	Peng et al. (2018)
* S. cerevisiae*	Amorpha-4,11-diene	Ergosterol-responsive promoters to regulate Erg9 transcription	350 mg/L	Yuan and Ching (2015)
* S. cerevisiae*	Lycopene	Growth-phase-dependent dynamic regulation	1.48 g/L	Su et al. (2020)
* S. cerevisiae*	α-Santalene	Dynamic regulation of *Erg9* expression with *HXT1*	92 mg/L	Scalcinati et al. (2012)
* S. cerevisiae*	Nerolidol	An auxin-inducible protein degradation system to decouple growth and production	3.5 g/L	Lu et al. (2021)
* S. cerevisiae*	Nerolidol	An endoplasmic reticulum-associated protein degradation of *Erg9p* to redirect flux towards sesquiterpene production	86% improvement	Peng et al. (2017)
* B. subtilis*	Menaquinone-7	A bifunctional and modular Phr-60-Rap-60-Spo0A QS system regulated by two endogenous promoters PabrB and PspoiiA	400-fold	Cui et al. (2019)
CRISPR interference (CRISPRi)				
* E. coli*	Isoprene, α-bisabolol and lycopene	Development of CRISPRi system for pathway regulation	2.6-, 10.6-, 8.0-fold increment, respectively	Kim et al. (2016)
* E. coli*	Isopentenol	Combinatorial knockdown of competing pathways with CRISPRi	98% improvement	Tian et al. (2019)
* P. putida*	Mevalonate	CRISPRi-mediated regulation of *glpR,* responsible for glycerol utilization	237 g/L	Kim et al. (2020)
* C. glutamicum*	Decaprenoxanthin	CRISPRi to identify regulatory genes for carotenoid biosynthesis	43- and ninefold	Göttl et al. (2021)
* C. glutamicum*	Squalene	CRISPRi-mediated repression of competing target genes	5.2-fold	Park et al. (2019)
* Synechocystis* sp. *PCC 6803*	Valencene	Downregulation of *crtE* with CRISPRi to decrease carotenoid production combined with fusion of *ispA* and *CnVS*	19 mg/g DCW	Dietsch et al. (2021)
* Methylorubrum extorquens*	Carotenoid	CRISPRi-mediated gene mining of phytoene desaturase as well as squalene-hopene cyclase gene repression	1.9-fold	Mo et al. (2020)
Promoter and RBS design				
* E. coli*	Geraniol	Optimization of GPP synthase with RBS	1119 mg/L	Zhou et al. (2015)
* E. coli*	β-carotene	Regulation of *atoB, mvaS,* and *Hmg1* with artificial regulatory parts, MI-46, M-37, and M1-93	51% increment	Ye et al. (2016)
* E. coli*	Viridiflorol and Amorphadiene	Transcription and translational optimization of enzymes	25.7 g/L and 30 g/L, respectively	Shukal et al. (2019)
* E. coli*	Amorphadiene	Combinatorial screening of RBS for translation of pathway enzymes	Fivefold increase	Nowroozi et al. 2014)
* E. coli*	Violaxanthin	RBS optimization of zeaxanthin epoxidase	231 µg/g DW	Takemura et al. (2019)
* E. coli*	α-Santalene	Promoter replacement to fine-tune the expression of iridoid synthase	599 g/L	Wang et al. (2021e)
* E. coli*	Steviol	Engineering of 5-UTR and N-terminal of pathway enzymes	38.4 ± 1.7 mg/L	Moon et al. (2020)
* E. coli*	Salicylate	A combinatorial screening of RBS sequences	123%	Qian et al. (2019)
* S. cerevisiae*	Sabinene	Downregulating *ERG20* with the glucose dependent weak promoter *PHXT*	19.4 mg/L	Jia et al. (2020)
* S. cerevisiae*	Squalene-type triterpenoids	Expression of CYP505D13 from *Ganoderma lucidum* on a yeast expression vector for squalene-type triterpenoids	3.28 mg/L, 13.77 mg/L, and 12.23 mg/L	Song et al. (2019)
* S. cerevisiae*	Linalool	Downregulating squalene production by replacing the endogenous ERG20 promoter with the sterol-responsive promoter *ERG1*	Threefold increment	Zhou et al. (2021)
* S. cerevisiae*	β-amyrin	Employing short synthetic terminators to regulate pathway	3.16-fold improvement	Ahmed et al. (2019)
* S. cerevisiae*	Lutein	Regulation of pathway enzymes with constitutive promoters as well as temperature-sensitive variant of transcriptional activator Gal4M9	N. A	Bian et al. (2021)
* S. cerevisiae*	Lycopene	Gal promoter screening	3.28 g/L	Shi et al. (2019)
* Y. lipolytica*	α-farnesene	Promoter optimization of Sc-tHMG1, IDI and OptFSLERG20	2.57 g/L	Liu et al. (2020d)
* Aspergillus oryzae*	Nepetalactol	Promoter replacement to fine-tune the expression of iridoid synthase	7.2 mg/L	Duan et al. (2021)
* Rhodobacter capsulatus*	Bisabolene	Promoter screening coupled with other pathway engineering strategies	9.8 g/L	Zhang et al. (2021b)
* Rhodobacter sphaeroides*	Pinene	RBS optimization coupled with fusion of geranyl diphosphate synthase and pinene synthase	N. A	Wu et al. (2021)
* C. glutamicum*	Astaxanthin	Combinatorial RBS, spacer, and start codon library for crtW and crtZ translation	0.4 mg/L/h	Henke et al. (2016)
* P. putida*	Mevalonate	Development of an inducible CRISPR activation (CRISPRa) system to regulate promoters	40-fold	Kiattisewee et al. (2021)
* Chlamydomonas reinhardtii*	Carotenoids	Overexpression of wild-type and mutant form of the plant regulatory protein ORANGE under a strong light inducible promoter	Two and threefold, respectively	Yazdani et al. (2021)
* Synechococcus elongatus UTEX 2973*	Limonene	Fine-tuning GPP synthase expression with synthetic RBS with varying translation rates coupled with *crtE* mutagenesis	16.4 mg/L	Lin et al. (2021)

QS systems that are completely orthogonal forestall the unexpected interference of the two components involved. Such systems are both signal and promoter orthogonal. Recently, the tra QS system from *Agrobacterium tumefaciens* and the las QS system from *Pseudomonas aeruginosa* were constructed into a complete orthogonality. To achieve this the EsaI was chosen to synthesize the inducer, *N*-(3-oxo-hexanoyl)-l-homoserine lactone (3OC6HSL), for the tra system. This system can be employed to metabolically regulate pathway genes (Jiang et al. 2020b). Other QS systems have recently been developed which could be applied to metabolically enhance cell factories’ productivity. For example, a homologous QS regulatory circuit system (hQSRC), a dual-input genetic controller that operates in three modes: (1) a constitutive model for high expression; (2) a tightly repressed mode, and (3) an inducible mode regulated by arabinose and autoinducer-2 was developed for QS-mediated protein expression in *Escherichia coli* (*E. coli*) (Hauk et al. 2020). Since QS controls processes that are touted as “expensive public goods” (Schuster et al. 2013), it will be costly and unproductive for a single cell to undertake such a process. Decoupling microbial biosynthesis into growth and production phases improves cell density that subsequently translates into high product formation. Since dynamic regulation predominantly supports a sufficiently dense population, there is a coordinate expression of the target gene when the population is at the large response. Also, dynamic regulation that ensures close interaction between cell density and gene expression could regulate pathway expression.

### CRISPR interference (CRISPRi)

Recent advancement in genome engineering has made it possible for biological researchers to directly delete, insert and modify DNA sequences of cells or organisms to elucidate their functions. A number of genomic editing technologies like zinc-finger nucleases (ZFN) based on eukaryotic transcription factors (Miller et al. 2007; Sander et al. 2011; Wood et al. 2011), transcription activator-like effector nucleases (TALENS) from *Xanthomonas* bacteria (Reyon et al. 2012; Sanjana et al. 2012; Wood et al. 2011; Zhang et al. 2011) and the most recent RNA-guided CRISPR–Cas nuclease system (Cho et al. 2013; Cong et al. 2013; Horvath and Barrangou 2010) have been employed in investigating genomic editing. Clustered regularly interspaced short palindromic repeats (CRISPR) with the CRISPR-associated (Cas) proteins system is RNA-mediated adaptive immune system in prokaryotes that protects them against bacteriophage and plasmid invasion (Barrangou et al. 2007; Barrangou and Marraffini 2014; McGinn and Marraffini 2016).

The CRISPR–Cas9 system has also been harnessed for genome regulation via the inactivation of the Cas9 protein (dCas9) (Qi et al. 2013; Schultenkämper et al. 2020). CRISPR interference (CRISPRi) or dead Cas9 (dCas9) is made possible by mutating the active region of the two domains of Cas9, RuvC and HNH, D10A and H840A, respectively, to attenuate the Cas9, yet retaining its binding ability (Bikard et al. 2013; Ma et al. 2015; Qi et al. 2013). This catalytically dead endonuclease hinders the transcriptional elongation of target genes. CRISPRi is an efficient promising tool to balance and modulate terpenoid production and cell growth as it precisely and predictably binds to fine-tune and repress target pathway genes instead of the traditional gene knockout strategy. This CRISPRi has been employed in pathway engineering to drive the flux towards the production of numerous terpenoids. More recently, CRISPRi-based system has been used to downregulate competing pathways for isopentenol (Tian et al. 2019), valencene (Dietsch et al. 2021). Table 2 provides a list of CRISPRi-mediated systems for regulating terpenoid pathways.

### Promoter and ribosome binding site (RBS) designs

Synthetic biology is a gene-combinatorial approach to combining pathway genes from different sources into a proposed metabolic pathway (Yadav et al. 2012). The conditional regulation of gene expression has for years been the object of scientific research. Gene expression can be tuned from transcription initiation, post-translational protein processing through the interaction between transcriptional and translational factors, repressors, activators, or enhancers. Post-transcriptional regulation always affects the level of translated proteins as the level of mRNA transcribed does not always correspond to translated proteins (Jeong et al. 2016; McManus et al. 2014). Promoters and RBS when with known functional characteristics, form an indispensable component of synthetic biology as they establish a baseline for transcription and translation of pathways to ensure optimized native and heterologous pathways (Lee and Trinh 2019). While the construction of well-characterized biological parts involves robust synthetic circuits, the incomplete characterization of the regulators in the construct result in an unstable output in a distinct genetic context. In a recent work to produce β-carotene in *E. coli*, Ye et al. regulated the expression of the endogenous acetyl-CoA acetyltransferase (*atoB*) as well as the exogenous enzymes 3-hydroxy-3-methyl-glutaryl-coenzyme A (HMG-CoA) synthase (*mvaS*) and HMG-CoA reductase (*Hmg1*) with artificial regulatory parts (MI-46, M-37, M1-93) possessing a characteristic constitutive strength and an RBS library, respectively. This ensured a 51% increment in β-carotene production (Ye et al. 2016).

**Fig. 4:**
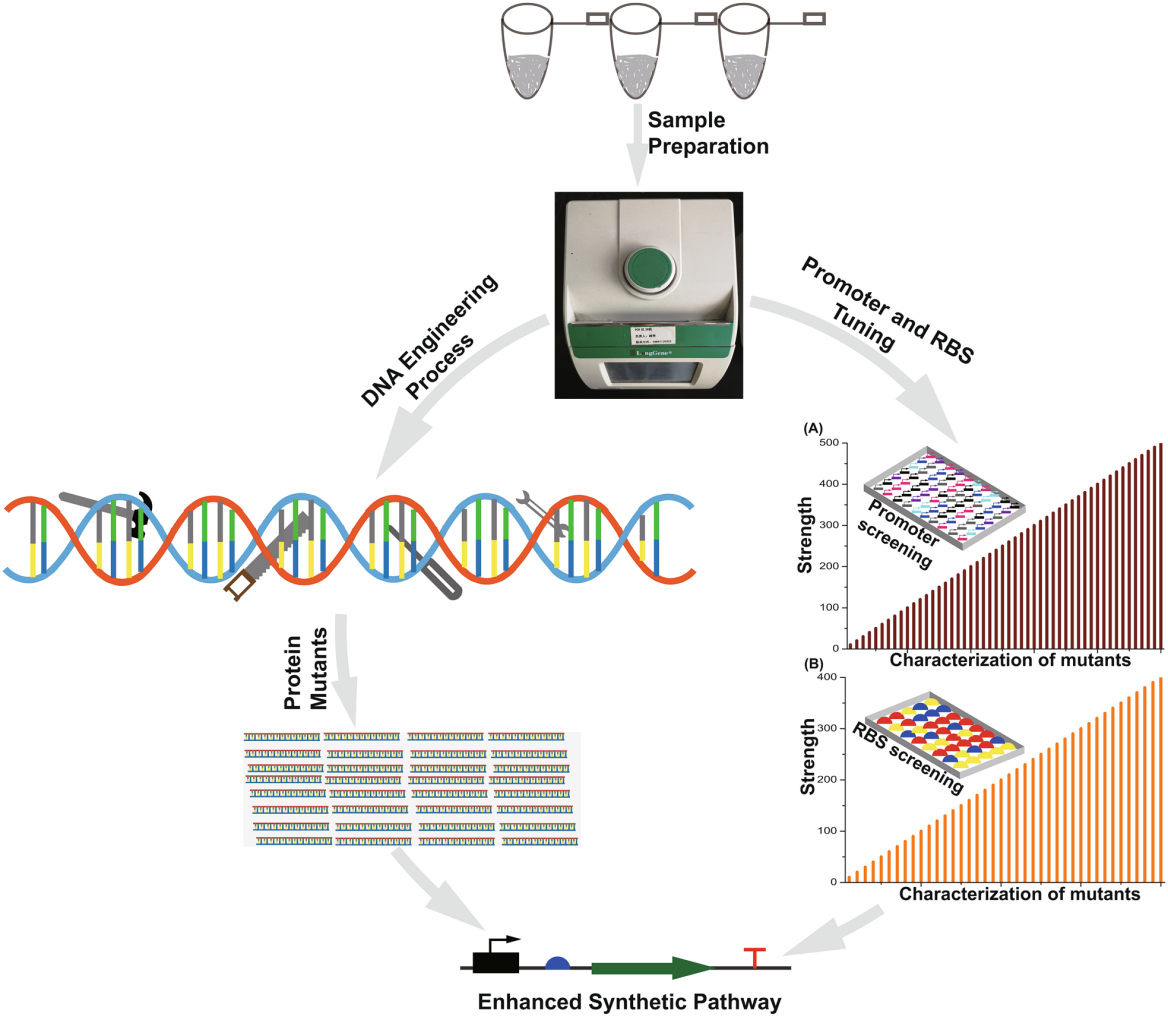
Schematic diagram for producing enzyme, promoter, and RBS library construction and screening. Directed evolution, site-directed mutagenesis, DNA shuffling can be employed to enhance enzyme and regulatory elements efficiency. Engineered promoters and RBS can be used to fine-tune biosynthetic pathways

Optimization of metabolic networks requires a quantitatively characterized pool of the regulatory elements for controlling the expression of target genes (Liu et al. 2018) (Fig. 4). However, several limitations hinder the generation and measurement of a large set of regulatory elements such as trial-and-error, making the processing time-consuming. Hence, a simple model was developed to determine the strength of a promoter based on RNA levels while RBS strength is determined by the efficiency of translation (Kosuri et al. 2013). To produce the sesquiterpene viridiflorol, the biosynthetic pathway was divided into three modules and regulated by a T7 promoter with varying strength. After a careful permutation to transcribe the modules to prevent the accumulation of intermediates and ensure high productivity, the best strain produced 283 mg/L of viridiflorol. RBS was also designed to control the translation of viridiflorol synthase (VS) which increased productivity to 511 ± 37 mg/L, ~ 50% increment (Shukal et al. 2019). In a similar related study, a combinatorial approach involving RBS with varying strength was used to fine-tune the production of amorphadiene. This led to a fivefold increase in amorphadiene accumulation with a subsequent reduction in toxic intermediate metabolites (Nowroozi et al. 2014). Violaxanthin is a carotenoid with numerous pharmaceutical and industrial applications. In the quest to enhance violaxanthin, Takemura et al. modified and designed RBS sequences to regulate *Capsicum annuum* zeaxanthin epoxidase (*CaZEP*), the enzyme responsible for catalyzing violaxanthin production from antheraxanthin. A 231 µg/g DW of violaxanthin was achieved (Takemura et al. 2019). Also, a convolutional neural network on cross-RBSs was used in fine-tuning biosensors’ dynamic range (Ding et al. 2020). This shows the importance of promoter and RBS engineering as powerful tools to enhance biosynthetic gene clusters found in transcriptionally silent natural products. The choice (inducible or constitutive) and strength affect the timing and expression level of target proteins. Hence, biological engineers focus on increasing size and complexity to precisely change the degree of expression of several different genes in a pathway (Han et al. 2019) (Table 2).

Regulating expression levels in multi-gene biosynthetic pathways represents a significant challenge for the building of microbial platforms due to a lack of effective control elements and resources (Wei et al. 2018). The cellular burden associated with multi-gene pathways could be minimized by altering each enzyme’s translation initiation rate (TIR) in the pathway of interest to maximizing titer and yield of the desired chemical (Kent and Dixon 2020). In a recent study to improve the supply of GPP for geraniol biosynthesis, the GPP synthase expression was optimized with RBS of increasing TIR. Optimizing the RBS strength resulted in a sixfold increment in geraniol production (Zhou et al. 2015). Though gene deactivation has been applied to block cellular stress response (Sharma et al. 2020), as well as amino acid supplementation to ensure efficient recombinant protein production (Kumar et al. 2020), various degrees of inducible and constitutive promoters have been employed to overcome the metabolic hurdles associated with transcription (Ye et al. 2016; Zhou et al. 2019). To avert the heterogeneities of cellular response that is associated with the use of chemical inducers and the need to regulate inducer concentrations, constitutive promoters have been used in the biosynthesis of the keto-carotenoids astaxanthin and canthaxanthin (Chou et al. 2019; Menin et al. 2019; Nora et al. 2019).

Machine learning has also been applied to predicting promoter sequences (Meng et al. 2017), identification of RNA/DNA binding proteins (Alipanahi et al. 2015), and predicting RBS sequence for improved chemical production (Jervis et al. 2019). In two related studies, machine learning was applied in the modeling pathway for limonene, bisabolene, and pinene synthesis (Dudley et al. 2020; Jervis et al. 2019). Native promoters could also be used to express biosynthetic pathways (Meng et al. 2016; Sengupta et al. 2019; Yuan and Ching 2015). However, they are frequently restricted by the host regulatory network. Synthetic promoters with enhanced strength and orthogonal to intrinsic control networks have been developed (Mordaka and Heap 2018; Redden and Alper 2015) to avert this problem. Since the titer of metabolites typically correlates with gene expression, synthetic biologists have sought the need to expand the synthetic biology toolbox by providing diversified regulatory parts for the construction of efficient endogenous or exogenous pathways (Table 2). However, the fitness of cell factories cannot be overlooked when designing pathway regulators (Bienick et al. 2014).

### Cellular tolerance engineering

*Saccharomyces cerevisiae* and *Escherichia coli* have been a “laboratory household name” as they continue to fascinate and bedazzle metabolic engineers and system biologists. They have been employed in building a fortified bio-economy. Nevertheless, as the demand for “go green” surges, metabolic engineers and system biologists have sought to enhance the cellular tolerance of these laboratory “workhorses” and other microorganisms to ensure improved cellular exportation for maximum product yield. Mitigation of toxicity effects of both end products and pathway intermediates is a major concern for designing cell factories for both research and industrial purposes. Isoprenoids and their associated intermediate products are of no exemption as excessive accumulation interferes with growth and metabolism. Hence, the need to explore membrane transporters for this purpose. The efflux system has been well studied and has been shown to enhance the biosynthesis of isoprenoids and other chemicals when effectively utilized. Efflux pumps or transporters are membrane proteins devoted to maintaining homeostasis by extruding toxic compounds from the intracellular environment (Jones et al. 2015; Putman et al. 2000). Transporter engineering ensures efficient exportation of compounds to alleviate product feedback inhibition and cytotoxicity (Eggeling and Sahm 2003). One possible alternative to prevent cellular toxicity at the early stages of fermentation is to express pathway genes under a tightly regulated promoter. The Gal promoter is a tightly inducible promoter that is induced by galactose and strongly repressed by glucose (Adams 1972). To alleviate *S. cerevisiae*’s toxicity to geraniol, pathway genes were expressed under the Gal promoter system (Jiang et al. 2017). Enhancing microbial tolerance coupled with other metabolic engineering has the propensity to increase the production of terpenoids in both model and non-model microorganisms. Some common transporter and tolerance engineering strategies that have been implicated in the biosynthesis of terpenoids are provided in Table 3.

**Table 3:** Cellular engineering strategies used to enhance the cell factory productivities

Strain	Product	Description	Outcome	Refs.
Cellular tolerance				
* E. coli*	Amorphadiene and kaurene	Overexpression of native AcrAB-TolC, MdtEF-TolC and the exogenous pump mexAB-OprM	118% and 104% improvement, respectively	Wang et al. (2013)
* E. coli*	Amorphadiene	Overexpression of lipopolysaccharide transport system	N. A	Zhang et al. (2016)
* E. coli*	α-Pinene	Overexpression of AcrB, AcrAB, and TtgB	1.9-fold	Niu et al. (2018)
* E. coli*	Sabinene	Overexpression of the genes *scpA*, *ygiZ* and *ybcK* via ALE	191.76 mg/L	Wu et al. (2020a)
* E. coli*	Isoprenol	Genome-wide knockout was employed to identify enzymes associated with isoprenol transport	NA	Wang et al. (2015)
* E. coli*	β-carotene	Development of an artificial membrane vesicle transport system	24-fold improvement	Wu et al. (2019)
* E. coli*	β-carotene	Engineering membrane bending proteins and membrane synthesis pathway	44.2 mg/g DCW	Wu et al. (2017)
* E. coli*	Lycopene	Membrane engineering via exogenous of and endogenous expression of *almgs*, *plsB, plsC* and *dgka,* respectively	36.4 mg/g DCW	Wu et al. (2018)
* E. coli*	Rainbow colorant	Membrane engineering via inner- and outer-membrane vesicle formation and cell morphology engineering	Varying	Yang et al. (2021)
* E. coli*	Squalene	Membrane engineering via membrane proteins overexpression	612 mg/L	Meng et al. (2020b)
* S. cerevisiae*	Astaxanthin	Atmospheric and room-temperature plasma as well as UV for strain improvement	404.78 mg/L	Jiang et al. (2020a)
* S. cerevisiae*	Crocetin	Development of a temperature-responsive strain coupled with chromosomal integration of pathway genes	139.64 ± 2.24 µg/g DCW	Liu et al. (2020e)
* S. cerevisiae*	β-carotene	Comparative proteomic and transcriptional analysis of ABC transporters	4.04-fold (secretion), 1.33-fold (intracellular)	Bu et al. (2020)
* S. cerevisiae*	Alkane	Exogenous expression of ABC transporters from *Y. lipolytica*	80-fold improvement	Chen et al. (2013a)
* S. cerevisiae*	Triterpenoids	Deletion of phosphatidic acid phosphatase (*PAH1*) for endoplasmic reticulum expansion	6-, 8-, 16-folds	Arendt et al. (2017)
* S. cerevisiae*	Geraniol	Immobilization of MVA pathway enzymes on yeast surface for in vitro fermentation	7.55 mg/L	Luo et al. (2021)
* Y. lipolytica*	α-, β-, γ-bisabolene	Exogenously expressing AcrB of the AcrAB-TolC system from *E. coli* and ABC-G1 from *Grosmania clavigera* under the constitutive promoter	2.7-, 8.5-, 1.2-fold, respectively	Zhao et al. (2021)
* Y. lipolytica*	β-carotene	Morphological engineering by deletion of *CLA4* and *MHY1* genes to convert mycelium form to the yeast form in addition with chromosomal integration	139% improvement	Liu et al. (2021c)
* Phaffia rhodozyma*	Astaxanthin	Combined atmospheric and room-temperature and UV mutagenesis	88.57 mg/L	(Zhuang et al. 2020)
* Phaffia rhodozyma*	Carotenoid	Application of magnetic field for improved cellular concentration	1146.39 ± 26.18 µg/L	(Silva et al. 2020)
Chromosomal integration				
* E. coli*	Astaxanthin	A plasmid-free strain	1.4 mg/g CDW	Lemuth et al. (2011)
* E. coli*	β-carotene	Integration of pathway genes	2.0 g/L	Li et al. (2015)
* E. coli*	β-carotene	Integration of two modules of MVA into the chromosome	26% improvement	Ye et al. (2016)
* E. coli*	Salvianic acid A	A plasmid-free strain	5.6 g/L	Zhou et al. (2017)
* E. coli*	Mevalonate	Integration of *atoB, mvaS, and mvaE* at *adhE* and *ldhA* loci	30 g/L	Wang et al. (2016)
* E. coli*	Bisabolene	Integration of sucrose utilizing operon and MVA pathway	Fivefold improvement	Alonso-Gutierrez et al. (2018)
* S. cerevisiae*	Geraniol	Integration of truncated geraniol synthase	236.34 mg/L	Jiang et al. (2017)
* S. cerevisiae*	Abscisic acid	Integration of the abscisic gene cluster coupled with plasmid expression	4.1-fold	Otto et al. (2019)
* S. cerevisiae*	Zerumbone	A multicopy integration of pathway genes	40 mg/L	Zhang et al. (2018a)
* S. cerevisiae*	Lycopene	Chromosomal integration of engineered *crtEB*	41.8 mg/g DCW	Hong et al. (2019)
* S. cerevisiae*	β-carotene	Chromosomal integration of β-carotene biosynthetic pathway genes from *Xanthophylomyces dendrorhous*	46.5 mg/g DCW	Fathi et al. (2021)
* S. cerevisiae*	8-hydroxygeraniol	Development of a plasmid-free strain	227 mg/L	Yee et al. (2019)
* S. cerevisiae*	Glycyrrhetinic acid and 11-oxo-β-amyrin	Chromosomal integration of glycyrrhetinic acid biosynthetic pathway in two representative strains, haploid and diploid	18.9 ± 2.0 mg/L and 108.1 ± 4.6 mg/L, respectively	Zhu et al. (2018)
* S. cerevisiae*	(−)-eremophilene	Genomic integration of an *Ocimum sanctum* sesquiterpene synthase	34.6 g/L	Deng et al. (2022)
* Y. lipolytica*	β-carotene	Integration of codon-optimized *carRA* and *carB* coupled with pathway optimization	1.7 g/L	Liu et al. (2021b)
* Y. lipolytica*	β-carotene	Multiple chromosomal integration of pathway enzymes under strong promoters	4 g/L	Gao et al. (2017)
* Y. lipolytica*	Isoprene	Genomic integration of codon-optimized isoprene synthase from *Pueraria montana* coupled with overexpression of pathway enzymes	~ 500 µg/L	Shaikh and Odaneth (2021)
* Bacillus subtilis*	Amorphadiene	Chromosomal integration of a fused amorphadiene synthase and green fluorescent protein	416 ± 15 mg/L	Pramastya et al. (2021)
Modularization				
* E. coli*	Isoprene	An inter- and intra-module of pathway	4.7-fold increment	Lv et al. (2016a)
* E. coli*	Taxadiene-5α-ol and Taxadiene	A multivariate-modular pathway	2400- and 15,000-fold increment, respectively	Ajikumar et al. (2010)
* E. coli*	Pinene	Modular co-culture of MVA pathway and a TIGR-mediated gene cluster	166.5 mg/L	Niu et al. (2018)
* S. cerevisiae*	Squalene and protopanaxadiol	Engineering of the endoplasmic reticulum as a special compartment triggered a global rewiring of metabolic pathway	71- and 8-fold, respectively	Kim et al. (2019)
* S. cerevisiae*	Isoprene	Dual regulation of the mitochondrial and endoplasmic reticulum compartments	2527 mg/L	Lv et al. (2016b)
* S. cerevisiae*	Ginsenoside compound K	Localization of pathway enzymes and metabolic intermediates to lipid droplets	5 g/L	Shi et al. (2021b)
* S. cerevisiae*	Squalene	Peroxisomal and cytoplasmic engineering	11 g/L	Liu et al. (2020a)
* S. cerevisiae*	Squalene	A combinatorial engineering of both cytoplasm and mitochondria to alleviate MVA pathway-related toxicity	21.1 g/L	Zhu et al. (2021)
* Y. lipolytica*	β-ionone	Enhancing cytosolic acetyl-CoA and MVA flux supply via modular engineering and fed-batch fermentation	0.98 g/L	Lu et al. (2020)
* Y. lipolytica*	Astaxanthin	Subcellular organelle compartmentalization of fused β-carotene ketolase and hydroxylase	858 mg/L	Ma et al. (2021)
* Pichia pastoris X33*	α-farnesene	Peroxisomal and cytoplasmic engineering	2.56 ± 0.04 g/L	Liu et al. (2021a)
* Bacillus subtilis*	Amorphadiene	Modularization of amorphadiene biosynthesis pathway including terpene synthase module, branch pathway module and central metabolic pathway module	116 mg/L	Song et al. (2021)

**Fig. 5:**
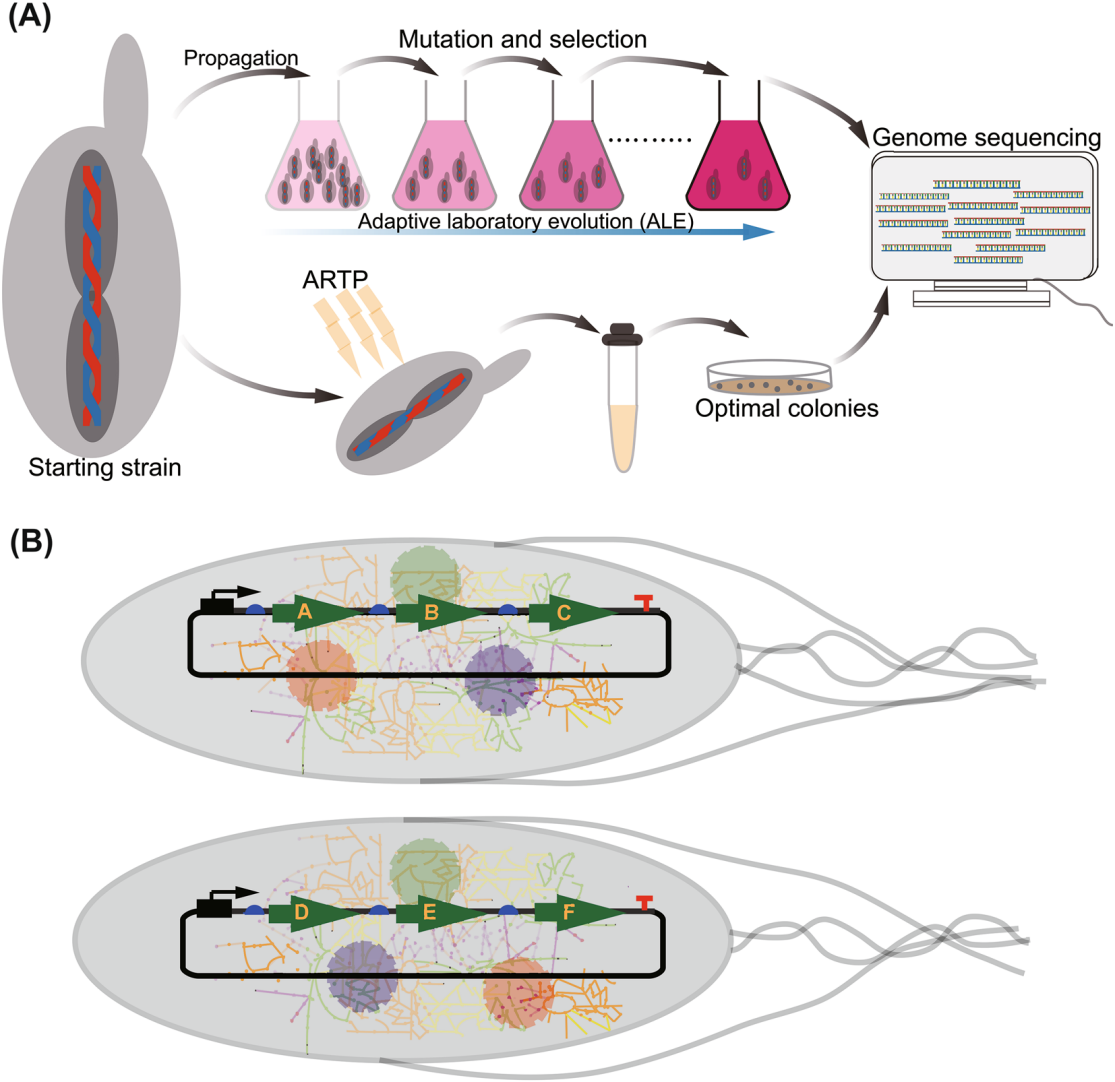
Schematic illustration of adaptive laboratory evolution (ALE), atmospheric and room-temperature plasma (ARTP), and modular co-culture. **A** Microorganisms are exposed to a desired selective mechanism and or environment for an iterative period enabling natural selection to optimize variants with enhanced fitness. Genome sequencing and transcriptome can be used to analyze mutant variants. **B** Modular co-culture engineering. Segregating pathway into modules ensures a holistic assessment of each part for efficient optimization and improvement

The overexpression of genes associated with efflux pumps has yielded positive results in the production of monoterpenes, sesquiterpenes, and diterpenes (Niu et al. 2018; Wang et al. 2013). Replacing the native promoter of *acrAB* in *E. coli* with the P_37_ strong promoter ensured improved tolerance to pinene (Niu et al. 2018). The cytotoxic effect of terpenoids and their precursors may be due to the damage to organelles, denaturation of proteins, disruption of biological processes, and damage to DNA and the lipid membrane (Nicolaou et al. 2010). Microorganisms have therefore developed a range of defensive strategies to react to these cytotoxic stresses, including overexpression of efflux pumps, activation of stress response genes, and changes in membrane structure (Ramos et al. 2002; Schalck et al. 2021). To build tolerant cell factories, evolutionary adaptation, whole-genome hybridization, genome shuffling, or random mutagenesis may be used, preceded by screening for enhanced variants under the appropriate conditions (David and Siewers 2015; Jullesson et al. 2015) (Fig. 5A). Adopting this strategy to improve the tolerance level of *E. coli* BL21 for the bicyclic monoterpene, sabinene, the strain was subjected to a gradually increasing concentration of sabinene to drive the evolution process. The mutant strain exhibited an 8.43-fold increase in sabinene production with total production reaching 191.76 mg/L. Transcriptome analysis revealed the overexpression of the genes methyl malonyl-CoA mutase (*scpA*), the protein that codes for the inner membrane (*ygiZ*), and the DLP12 prophage family (*ybcK*) that have been touted to enhance terpene tolerance (Wu et al. 2020a). Bu et al. adopting both comparative proteomics and transcriptional analysis identified five suitable ABC transporters that efficiently transport β-carotene in *Saccharomyces cerevisiae*. This ensured a 4.04- and 1.33-fold increase in the secretion and intracellular production of β-carotene, respectively (Bu et al. 2020).

In the process to develop solvent tolerance, Gram-negative bacteria develop: (i) an effective change in the composition of membrane fatty acids and phospholipid headgroups; (ii) vesicles containing toxic substances and (iii) toxic organic solvents are exported to the extracellular environment through a resistance-nodulation-cell division (RND) family, an active efflux pump that is energy-dependent (Ramos et al. 2002). The inability of industrial microorganisms to excrete metabolites hinders normal cellular function, which further affects productivity. The biosynthesis of hydrophobic compounds poses a major threat to cell physiological functioning. Carotenoids are large hydrophobic molecules that are not easily excreted by microbial transport systems resulting in intracellular accumulation. An artificial membrane vesicle transport system (AMVTS) was constructed that utilizes membrane lipids to transport hydrophobic compounds. The application of the AMVTS in a β-carotene hyper-producing strain led to a 24-fold increase of secreted β-carotene and a 61% increase in specific production from 27.7 to 44.8 mg/g DCW (Wu et al. 2019). The plasma membrane ABC efflux system is one of the many promising areas for building suitable cell factories for industrial application. To improve the biosynthesis of terpenoids, an improvement in terpenoid-associated transporters must be considered. Moreover, the efflux system is known to be broadly ranged. However, solvent-specific exporters are also known to exist. Hence, it is imperative to carefully study the efflux system to elucidate how they recognize and transport molecules to help engineer product-specific pumps.

### Chromosomal integration

Plasmids have been used as an expression system for both endogenous and exogenous pathway genes as it is easy to use or manipulate and incorporate into the host strain, portable, and high copy numbers per cell (Gu et al. 2015; Karim et al. 2013). However, they come with segregational and structural instability, aside from the requirement of antibiotics and sometimes inducer chemicals, resulting in a high cost of production (Friehs 2004). The plasmid-mediated expression system also tends to increase the metabolic burden, especially when using high-copy plasmids. This is due to the channeling of energy towards the keeping and replication of plasmids resulting in poor growth of host cells, leading to low productivity (Wu et al. 2016). Genomic integration provides a way to stably insert pathway genes in the chromosome of desired hosts, which could function with or without antibiotics and inducers. Capitalizing on its stable expression of pathway genes, chromosomal integration was used to enhance geraniol production when a truncated geraniol synthase was integrated into *S. cerevisiae*’s genome. This resulted in a 23% increment in production, 236.34 mg/L (Jiang et al. 2017).

Plasmid-free cell factories have also been employed in the production of astaxanthin (Lemuth et al. 2011), β-carotene (Li et al. 2015), and many other terpenoids found in Table 3. However, in most cases, the total yield does not meet the industrial requirements. To enhance the expression of pathway genes on the chromosome, several strategies including (Ou et al. 2018): (i) increasing copy numbers of the target gene; (ii) chromosomal loci for integration, and (iii) optimization of the target gene on the chromosome through static and dynamic regulators, have been adopted. The ability to support the expression of pathway genes differs significantly from different chromosomal loci, and these loci ought to be highly expressed, conserved, well-characterized as well as non-essential (Bryant et al. 2014; Yin et al. 2015). To accomplish this, different strategies and methods have been used to stably integrate target genes into chromosomal loci while addressing the issue of copy numbers. To construct an industrial strain for the production of zerumbone, a multicopy integration of the pathway enzymes including cytochrome P450 and a type III membrane protein (*ICE2*) were integrated into the *S. cerevisiae* chromosome resulting in a 134-fold 8-hydroxy-α-humulene production. A subsequent multicopy integration of the zerumbone synthase variant (*ZSD1*^S144A^) yielded 20.6 mg/L of zerumbone and 40 mg/L in a 5-L bioreactor (Zhang et al. 2018a). Being a precursor for the biosynthesis of isoprenoids (Liu et al. 2019; Marsafari and Xu 2020), mevalonate is also an important industrial component (Xiong et al. 2014). To alleviate the metabolic burden and genetic instability associated with the plasmid expression system while reducing the cost of production to ensure industrial application, two copies of the mevalonate pathway (*atoB*–*mvaS*–*mvaE*) under the expression of a strong constitutive promoter was integrated into *E. coli* chromosome to replace the *adhE* and *ldhA* genes for mevalonate production. Coupled with another pathway engineering (the deletion of *sucA* and *atpFH* genes), the final strain produced 30 g/L of mevalonate from 61 g/L glucose in a fed-batch fermentation (Wang et al. 2016). The flippase from the yeast 2-μm plasmid was effectively used to increase the product output and stability using chromosomal integration of gene(s) with multiple copies (CIGMC) (Gu et al. 2015). Another novel strategy involving *λ* and *φ*80 bacteriophage site-specific recombination and integration systems have also been used to insert operons of 7.5 and 14 kb into the chromosome with subsequent duplication of target genes (Igonina et al. 2020). Other strategies include chemically inducible chromosomal evolution (CIChE) system (Tyo et al. 2009), clonetegration—a one-step cloning and chromosomal integration of DNA (St-Pierre et al. 2013), a homologous recombination-based method that involves a linear DNA fragment flanked by homologous arms (Storici et al. 2003). A delta integration CRISPR–Cas9 method that ensures a multicopy, highly efficient, one-step and marker-less integration of DNA constructs at the delta sites of the *S. cerevisiae* chromosome was also developed. This method ensures that fragments spanning from 8 to 24 kb are seamlessly integrated (Shi et al. 2016).

The creation of chromosomally engineered strains is suitable for commercial production, but the relatively low expression on the chromosome has sometimes resulted in inefficient production of target chemicals. However, the expression of exogenous pathway genes using chromosomal integration is highly stable compared to the plasmid-mediated expression system. In line with developing highly efficient platforms for terpenoid production for industrial usage, targeted chromosomal engineering can be coupled with pathway optimization for a precursor supply and an efficient dynamic regulatory system.

### Modularization of synthetic pathways

Metabolic pathways involving a large number of genes are notably associated with flux imbalance. Grouping the pathway into suitable modules represents an efficient solution to addressing this issue (Pfleger and Prather 2015; Smanski et al. 2014). Modular co-culture engineering, which involves the partition of pathways into modules and integrating them into separate expression hosts, has also been used to synthesize compounds (Fig. 5B). This system has some advantages of (i) lowering each host metabolic burden; (ii) delivering a variety of cellular conditions in which the various regulatory genes can act; (iii) limiting unintended interaction between various pathways; (iv) adjusting the strain-to-strain ratio to balance the biosynthetic pathway among independent pathway modules; (v) maximizing the performance of complex structures with multiple active substrates; and (vi) facilitating the biosynthesis of a variety of target compounds in a plug-and-play manner (Zhang and Wang 2016). The terpenoid pathway involves several multi-step enzymatic reactions. Hence, constructing cell factories often requires extensive metabolism regulations to ensure an enhanced synthesis of the desired end products. To achieve this goal, several impediments such as (i) tuning the metabolic flux towards target products; (ii) balancing and coordinating corresponding pathways and enzymes to prevent pathway perturbations; (iii) increasing the supply of precursors especially if the strain is growing on glucose to prevent the Crabtree effect; (iv) implementing strategies to optimize the entire pathway (Qin et al. 2015), have to be addressed. To enhance the biosynthesis of isoprene, the isoprene synthetic pathway was divided into two modules; the upstream endogenous MEP pathway and the downstream isoprene pathway made up of the isoprene synthase. An intra-module protein engineering strategy was used to improve the rate-limiting *dxs/dxr/idi*, while inter-module engineering involving promoter replacement and inducer adjustment was conducted to enhance isoprene synthesis. The final strain achieved a 4.7-fold increment in isoprene as compared to the wild-type strain (Lv et al. 2016a).

In a multi-enzyme synthetic pathway, simple overexpression of rate-limiting enzymes is often associated with an imbalanced pathway that results in the accumulation of toxic intermediate metabolites (Sivy et al. 2011). Recently, a multi-modular engineering approach involving alleviating feedback inhibition, and other pathway engineering strategies were adopted for tyrosol and salidroside overproduction in *S. cerevisiae* (Liu et al. 2020b). The multidimensional heuristic process (MHP) is a modular pathway optimization approach that assembles and screens multiple repositories of clearly defined transcription factors as well as main enzyme variants in a high-dimensional combinatorial approach to create high-producing strains (Zhang et al. 2018b). This system was used to produce nerolidol, linalool, and astaxanthin from *E. coli* by partitioning the pathway into three and four modules, respectively (Zhang et al. 2018b). In another related study, the complete β-ionone pathway was divided into three modules: module one responsible for enhancing acetyl-CoA supply comprises the exogenous phosphoketolase from *Bifidobacterium bifidum* and phosphotransacetylase from *Bacillus subtilis*; module two contains the endogenous MVA pathway; while the module three consists of the exogenous β-ionone module made up of phytoene dehydrogenase (*carB*), phytoene synthase/lycopene cyclase (*carRP*) and carotenoid cleavage dioxygenase (*ccD1*). These modules were sequentially divided into *Yarrowia lipolytica*. After pathway optimization augmented with medium and fed-batch optimization, 0.98 g/L of β-ionone was produced after 17 days (Lu et al. 2020). In other instances, in the yeast cells, terpenoid pathways have been segregated into the mitochondria, peroxisomes, endoplasmic reticulum, and cytoplasm compartments to ensure precursor availability for target product synthesis. Table 3 lists several conventional modular approaches that have been used in developing industrial microorganisms.

Modularization of pathways results in a targeted rectification of bottlenecks. Segregating the multi-gene pathway is an efficient way to optimize the expression of a biosynthetic pathway. This provides an effective way to manipulate individual expression levels as it reduces pathway complexity and provides avenues for future uncertainties.

## Future perspective for designing efficient microbial platforms

Synthetic biology and metabolic engineering and have demonstrated great capability in ensuring the engineered platforms for the biosynthesis of terpenoids and other essential compounds. The tremendous structural diversity of terpenoids resulting in the vast chemicals for various industries can be linked to terpene synthases’ promiscuous behavior (Fig. 1). Taking into account the biochemical and physiochemical properties of terpenoids, process engineering is a requisite factor when designing cell factories for isoprenoids production. This will allay the inhibitory and toxic effects of isoprenoids and their precursors. Terpenoids inhibition effect is a result of metabolite cytotoxicity in production strains above its threshold. High concentrations of terpenoids and their intermediate products impede cell growth with a subsequent effect on total production. Hence, a suitable method to remove the metabolites at lower concentrations is to enhance the productivity of the biocatalyst. In situ product removal (ISPR) has enhanced higher titers as it prevents product accumulation in culture media and interactions with cells (Alonso-Gutierrez et al. 2013; Brennan et al. 2012; Dong et al. 2020b; Rolf et al. 2020; Schewe et al. 2015). Hence, the need to investigate suitable ISPR mechanisms. The implementation of ISPR is a technical approach to tackling toxicity-associated low productivity and minimizing product loss (Freeman et al. 1993; Salas-Villalobos et al. 2021). Compounds’ hydrophobicity, molecular weight, charge, volatility, and specific binding properties play a major factor in the choice of a compound for the ISPR approach (Freeman et al. 1993). In the two-liquid phase system, one common separation method, two aqueous solutions form a fine emulsion which is correlated to the agitation speed of the reactor. Since the receiving phase is a hydrocarbon, its biocompatibility or toxic effect on the cell factory should be considered. This can be computed with its log *P*_oct_ (Laane et al. 1987). To expedite the downstream extraction of astaxanthin while taking into consideration the techno economic analysis, an alternative method was established in *Haematococcus pluvialis* that involves extraction with ethyl acetate from zoospores when growth conditions are restored. This method yielded an 85% extraction rate with 3.5 g of astaxanthin oleoresin produced (Bauer and Minceva 2021). Recently, a pulsed electric field technology, an electroporation method of treating cell biomass before subsequent treatment with traditional solvents or supercritical carbon dioxide was applied to improve the extraction of carotenoids (Martínez et al. 2018, 2020; Saini and Keum 2018). In the downstream processing of industrial-scale bioprocesses, the organic phase becomes a significant practical concern. Microorganisms can also be engineered to enhance their resilience on these hydrophobic chemicals or through the use of solvent-tolerant microbes.

Because all essential information is derived via experimental data rather than meticulously obtained and introduced by domain specialists, a machine learning-based technique allows for the speedier construction of predictive pathway dynamics models. Machine learning (ML), the application of data-driven algorithms could be used to predict the contribution of each particular gene to a specific trait that may be used to analyze, optimize, and develop metabolic or neural networks (Lawson et al. 2021; Mowbray et al. 2021), to improve microbial growth and product synthesis. For several terpene synthases, prenyltransferases that lack structural data, molecular modeling via ML could be used to predict the 3D structures of enzymes, which can then be combined with enzyme–substrate docking studies to enhance several properties such as stability, activity, and specificity (Mazurenko et al. 2020; Singh et al. 2021; Yang et al. 2019). ML can be useful in analyzing the effectiveness of microbial factories via transitional genome-scale modeling, predicting cell phenotypes, and characterizing cell growth (Culley et al. 2020). ML-based modeling has the potential to successfully help design efficient cell factories in the future without knowing comprehensive metabolic regulation pathways. Nevertheless, high-quality quantitative data in multiple situations are required to help address the issues of enzyme engineering, transcription factor binding sites, translation control, ribosomal binding sites, and growth optimization (Helmy et al. 2020).

**Fig. 6:**
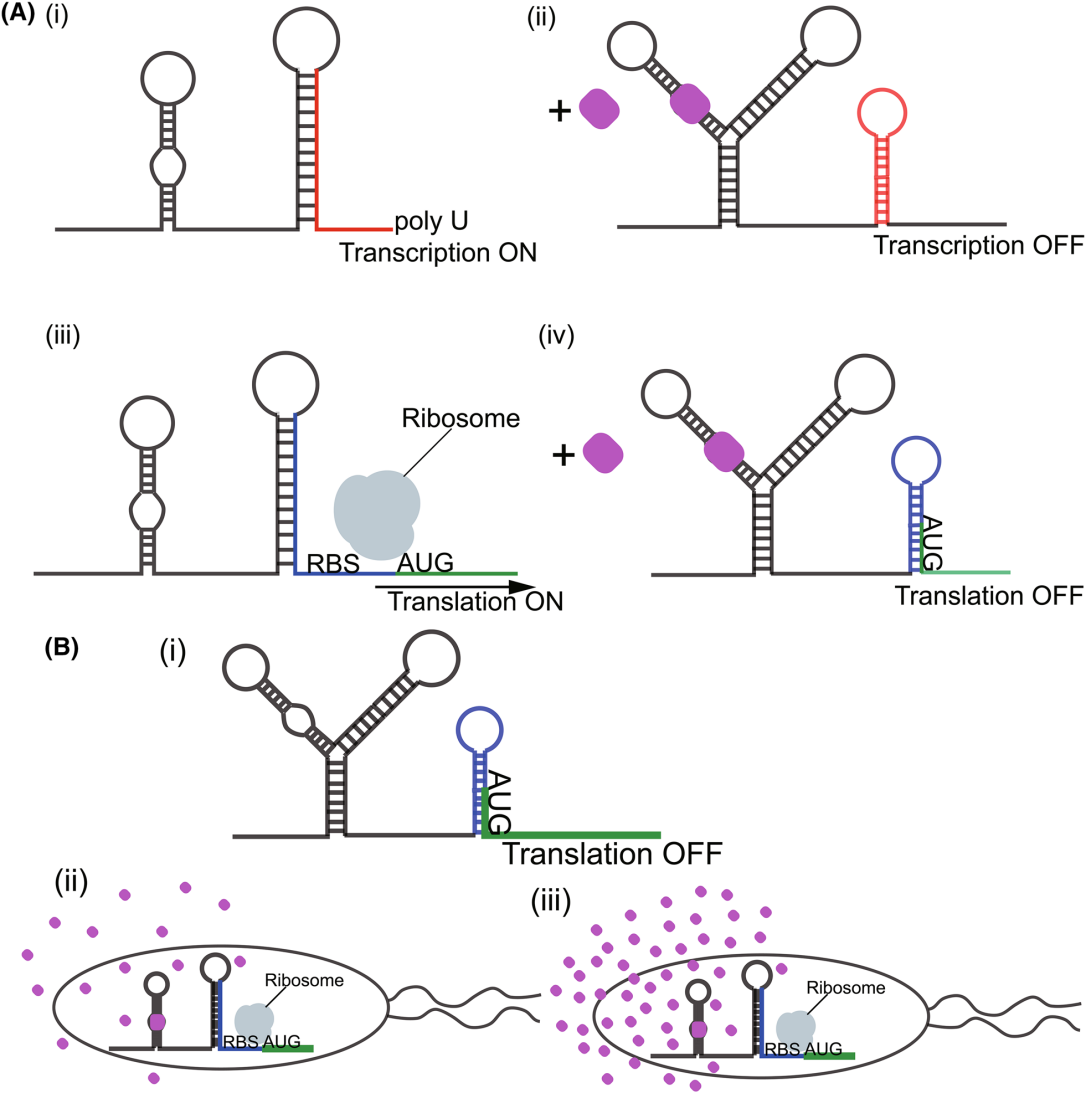
Mechanism of action of riboswitches. **A (i, ii)** Binding of ligand to a riboswitch triggers the formation of a hairpin loop that terminates transcription. **A (iii, iv)** Binding of ligand to a riboswitch generates the formation of a helix that sequesters the RBS to inhibit the translational process. **B (i, ii, iii)** Application of riboswitches as biosensors. Riboswitches can be linked to colorimetric reporters (for example GFP) to screen for high-producing strains depending on the concentration of the compound of interest. There is a high expression of the reporter gene when the concentration of a ligand is high **(B ii)** and vice versa

Analysis of metabolic flux is a significant indicator of productive cells. However, cellular metabolites are mainly measured through LC–MS and or GC–MS, a time-consuming throughput for screening in microbial engineering. The inability to precisely quantify and regulate metabolite concentration-related genetic variants especially for most terpenoids, becomes a bottleneck in metabolic engineering (Liu et al. 2017). Metabolite biosensors have gained tremendous recognition in metabolic engineering as these RNA sensors or genetically encoded proteins interact with metabolites to generate detectable phenotypes through the modulation of protein expression (Liu et al. 2015). In addition, due to their quick, precise, and effective mode of action and ease of processing and engineering, biological sensors can respond to different environmental stimuli creating a molecular network. Transcriptional factor-based biosensors are powerful tools that can be used as a high-throughput screening method to develop high-producing strains (Yu et al. 2019) as they present otherwise-obscured intracellular states to a screenable output (Fig. 6B). One such example is a riboswitch which can be turned on and off to regulate expression (Page et al. 2018) (Fig. 6A). In a recent related study, the carotenoid biosynthesis regulator, *crtR*, in *C. glutamicum* was engineered to measure the intracellular GGPP concentration during growth (Henke et al. 2020). These sensors could be employed as a high-throughput to screen for isoprenoid-producing strains. Not only do biosensors serve as a high-throughput screening method, but they can also be engineered to function as a dynamic tool. Though it has found its application in developing strains for terpenoid production, adaptive laboratory evolution (ALE), a laboratory “natural selection” process as well as UV and atmospheric and room-temperature plasma (ARTP) holds a brighter prospect of improving cell’s performance, product and intermediates tolerance, and growth rate (Fig. 5A). This random mutation and selection process causes a global disturbance in the genome that will provide additional insights into the regulatory and metabolic circuitry, subsequently providing a platform for developing high-performing strains. Mutants can then be thoroughly screened by a suitable high-throughput method.

An emerging synthetic pathway that is demonstrating as a promising alternative to the inherently constrained native MVA and MEP pathways termed isopentenyl utilizing pathway (IUP) is gaining grounds. This non-canonical pathway that utilizes isopentenol isomers or prenols as substrate is made up of four genes for monoterpenes synthesis as against the lengthy enzymatic MVA–MEP pathways (Ward et al. 2019). This pathway has been implicated in cell free synthesis of mono-, sesqui-, di-terpenes (Ward et al. 2019). IUP has also been employed in the biosynthesis of various types of terpenoids including linalool (Ferraz et al. 2021), geraniol (Clomburg et al. 2019), nerol, citronellol, lycopene (Chatzivasileiou et al. 2019; Lund et al. 2019; Luo et al. 2020). In addition, a lepidopteran mevalonate (LMVA) pathway has been constructed in *E. coli* by linking the LMVA pathway with the promiscuous phosphate, *NudB*. Deletion of the endogenous thiolase genes yielded 390 mg/L of C6-isoprenol (Pang et al. 2021). Likewise, construction of a pyruvate dehydrogenase (*PDH*) by-pass coupled with gene deletions for enhanced acetyl-CoA flux through the MVA pathway resulted in 2.23 g/L limonene production in *S. cerevisiae* from a fed-batch shake-flask fermentation (Zhang et al. 2021a). Employing *Acinetobacter baylyi* ADP1 that catabolizes lignin-derived aromatic substrates couple with gene inactivation and fermentation optimization, Arvay et al. (2021), were able to produce 1014 mg/L of mevalonate through the β-ketoadipate pathway.

Other key strategies include the identification of key regulatory enzymes that could ensure the upregulation of key pathway enzymes for the accumulation of metabolites. This strategy was used in the overproduction of astaxanthin in *Xanthophyllomyces dendrorhous* when 6-benzylaminopurine was overexpressed (Pan et al. 2020). Also, overexpression of key pathway enzymes have yielded a 1.16-fold increment of sabinene in *S. cerevisiae* (Jia et al. 2020), 731.18 mg/L of squalene in *Y. lipolytica* (Tang et al. 2021) and 180 mg/L of bisabolene in *Synechocystis* sp. PCC 6803 (Rodrigues and Lindberg 2021). Pathway engineering via deletion of competing pathways in the central carbon pathway for acetyl-CoA accumulation while increasing the availability of NADPH has also proven positive as seen in β-carotene production (Wu et al. 2020b). Also, a reduction in lipid biosynthesis through the inactivation of diacylglycerol acyltransferases produced 22.8 g/L of β-farnesene in *Y. lipolytica* (Shi et al. 2021a), whereas inactivation of phytoene synthase (*dr0862*) in *Deinococcus radiodurans* yielded 3.2 ± 0.2 mg/L of pinene (Helalat et al. 2021). Optimization of fermentation parameters, fermentation medium, and terpenoid pathway could improve biomass production while increasing the synthesis of target metabolites (Dai et al. 2021; Liu et al. 2020c, d; Lv et al. 2020; Walls et al. 2020). More so, microorganisms or host organisms with endogenous high-flux isoprenoid pathways, as well as highly tolerant strains like *P. putida* (Ramos et al. 2015) that have stringent responses to organic solvents could be explored for the biosynthesis of terpenoids (Ankenbauer et al. 2020; Mishra et al. 2020).

## Conclusion

Enormous advances have been accomplished in the past decade to engineer microbial platforms for terpenoid biosynthesis; however, these strains still face challenges owing to the intricacy of the terpenoid pathway and tight regulatory networks. Here, we demonstrate the viable approaches for improving the biosynthesis of terpenoids. Overall, efforts towards ensuring suitable microbial platforms have been discussed here for the industrial production of terpenoids. We urge metabolic engineers and synthetic biologists that with the increasing developments in the field, more progress should be made in designing chassis for the biosynthesis of terpenoids.

## Data Availability

All datasets used and analyzed are available on reasonable request.

## Abbreviations

MVA: Mevalonate
MEP: 2-C-Methyl-d-erythritol 4-phosphate
IPP: Isopentenyl diphosphate
DMAPP: Dimethylallyl diphosphate
QS: Quorum sensing
AHL: Acyl homoserine lactose
RBS: Ribosome binding site
LC–MS: Liquid chromatograph–mass spectrometer
GC–MS: Gas chromatograph–mass spectrometer
UTR: Untranslated region
RND: Resistance nodulation-cell division
CIGMC: Chromosomal integration of gene(s) with multiple copies
CIChE: Chemically inducible chromosomal evolution
AMVTS: Artificial membrane vesicle transport system
MHP: Multidimensional heuristic process
CRISPR: Clustered regular interspaced palindromic repeats
CRISPRi: CRISPR interference
ALE: Adaptive laboratory evolution
ARTP: Atmospheric and room-temperature plasma
ISPR: In situ product removal (ISPR)
ML: Machine learning
